# “Is eat, sleep, console the key to safer and faster discharge for newborns with neonatal opioid withdrawal syndrome?”

**DOI:** 10.1038/s41372-025-02216-1

**Published:** 2025-02-06

**Authors:** Ahmed Ismaiel, Nisha Sheikh, Deborah Campbell

**Affiliations:** 1 Division of Neonatology, Children’s Hospital at Montefiore, Albert Einstein College of Medicine, Bronx, NY USA; 2 Pediatric Department, Children’s Hospital at Montefiore, Albert Einstein College of Medicine, Bronx, NY USA

**Keywords:** Paediatrics, Drug therapy

## Manuscript Citation

Young, L. W., Ounpraseuth, S. T., Merhar, S. L., Hu, Z., Simon, A. E., Bremer, A. A., … & Devlin, L. A. (2023). Eat, sleep, console approach, or usual care for neonatal opioid withdrawal. New England Journal of Medicine, 388(25), 2326–2337. (1)

### Question

Does the Eat, Sleep, Console approach to neonatal opioid withdrawal decrease the number of days until infants are medically ready for discharge without adverse outcomes compared to the Usual Care approach?

## Methods

### Design

The trial design involved 26 U.S. hospitals participating in the ACT NOW Collaborative multicenter, stepped-wedge, cluster-randomized, controlled trial. An independent data and safety monitoring committee provided oversight of trial conduct [1].

### Patients

Infants born at 36 weeks gestation or later, either delivered at or transferred to a trial site within 60 h of birth, were included in the study. Care was provided across diverse academic and community hospitals nationwide. To minimize bias, the authors ensured site variation by selecting locations with differing numbers of opioid withdrawal cases, treatment unit types, and nonpharmacologic interventions. Screening criteria required evidence of antenatal opioid exposure and the need for opioid withdrawal treatment.

### Randomization

The 26 sites were stratified based on the proportion of infants who were being treated pharmacologically before trial initiation. Within each strata, sites were randomized into one of eight blocks. Each block of sites was then randomized to the time of transition to the Eat Sleep Console (ESC) approach.

### Intervention

Before the transition period, sites maintained their usual care practices, including assessments with the Finnegan (FNAST)/modified Finnegan tool (MFSS) and any pre-existing non-pharmacologic interventions. During the transition, each site underwent staff training for the ESC approach. With the start of the intervention phase, the ESC approach replaced Finnegan scoring. Throughout the trial, sites continued their established pharmacologic treatment and discharge practices.

## Outcomes

### Primary outcome

Time from birth to medical readiness for discharge. Medical readiness criteria were age of at least 96 h, at least 48 h without opioid medications, at least 24 h without respiratory support and on full oral feeding, and at least 24 h of feeding with maximum caloric density.

### Secondary outcomes

Main outcomes included receipt of pharmacological treatment, length of hospital stay (LOS), and safety outcomes that encompassed an in-hospital composite safety measure (e.g., seizures, accidental trauma, or respiratory insufficiency secondary to pharmacologic treatment), a 3-month postnatal age composite safety measure (e.g., acute health care utilization, emergency department visit, or hospital readmission), and a composite critical safety outcome from discharge through 3 months of age (e.g., non-accidental trauma or death).

### Statistical analysis

Enrollment of 864 infants provided 90% power to detect a 4-day difference in the average time from birth to medical readiness for discharge between groups, assuming an intraclass correlation coefficient of 0.25, which showed how similar the outcomes within each group were and a cluster autocorrelation coefficient of 0.8 were used to account for within- and between-period correlations. The analysis adhered to the intention-to-treat principle, using a two-sided significance level of 0.05. A generalized linear mixed model with an appropriate distribution (negative binomial, Poisson, gamma, or Gaussian) and appropriate link (identity or log) was used to model the primary and secondary outcomes. Each model included intervention and time as fixed effects and a site-specific random intercept to account for the clustering of infants within sites. The heterogeneity of the treatment effect across sites and trial periods was assessed by including interaction fixed effects. Infants who had been discharged before meeting the trial definition of medical readiness for discharge were not included in the primary analysis of the primary endpoint and so a frailty model time-to-event analysis was conducted as a sensitivity analysis. This was conducted to assess whether the heterogeneity in intervention effect was present.

## Results

### Patients

1874 participants underwent the screening process, and 1305 were enrolled. The proportion of Hispanic mothers and those residing in metro areas were different between randomized groups, with more Hispanic mothers in the usual care group and more metro area residents in the ESC group. The number of non-Hispanic Black and Hispanic infants was overrepresented in the usual care group when compared to the reported national rates of maternal substance use disorder in pregnancy. In the ESC group, there was an under-representation of Hispanic infants. However, the sites were representative of the geographical diversity overall.

#### Primary outcome

The study found that amongst infants who met criteria for being medically ready for discharge, infants in the ESC group had a shorter mean LOS compared to the usual care group (8.2 vs. 14.9 days, with an adjusted mean difference of 6.7 days; 95% CI, 4.7–8.8). The rate ratio was 0.55 (95% CI, 0.46–0.65; *p* < 0.001), so a 45% reduction in LOS. While the results remained consistent across study periods, there was evidence that the treatment effect varied across the sites.

#### Secondary and safety outcomes

The mean LOS in the ESC group was 7.8 days versus usual care which averaged 14.0 days (mean difference, 6.2 days; 95% CI, 4.6–7.7; rate ratio, 0.56; 95% CI, 0.49–0.64). 52% of infants in the usual care group received opioid treatment vs. 19.5% in the ESC group (absolute difference, 32.5 percentage points; relative risk, 0.38; 95% CI, 0.30–0.47). Infants in the ESC group had the same risk of adverse outcomes 3 months after discharge compared to the usual care group, 16.1%, and 15.8%, respectively; relative risk, 1.02; 95% CI, 0.71–1.47.

#### Study conclusion

Compared to infants assessed with FNASS/MFSS, functional assessment of infants with neonatal opioid withdrawal syndrome significantly decreased the length of stay by 45% amongst infants who met the criteria for medical readiness for discharge. The ESC approach also reduced receipt of opioid treatment by 30% and did not increase adverse outcomes.

## Commentary

Neonatal abstinence syndrome (NAS) is a growing clinical concern characterized by a constellation of withdrawal symptoms in newborns exposed to prenatal opioids [2]. The incidence of NAS has surged in recent decades, placing a significant burden on healthcare systems [3]. Traditional management focused on partially subjective FNASS/MFSS assessments has led to extensive pharmacologic intervention. The Eat, Sleep, Console method emphasizes functional assessments to support infant neurobehavior and reduce opioid treatment [4].

The incidence of NAS in the United States has risen nearly fivefold, from 1.2 per 1,000 births in 2000 to 7.3 per 1000 births in 2017 [5, 6] in conjunction with the burgeoning opioid epidemic, leading to more infants requiring medical attention for withdrawal symptoms. The average length of stay for infants treated for NAS based on the use of the FNASS/MFSS is approximately 23 days. The combined effects of prolonged hospitalizations, intensive monitoring, and treatment needs place an estimated annual burden of $1.5 billion on the healthcare system [7].

Over the past 50 years, the management of NAS has evolved significantly. Initially, the FNASS, developed in the 1970s, guided pharmacologic treatment based on 21 clinical signs of withdrawal [8]. Recently, the American Academy of Pediatrics (AAP) recommended a two-tiered approach emphasizing first-line non-pharmacologic care. Dr. Matthew Grossman at Yale University introduced the ESC method in 2014, which focused on the infant’s ability to eat, sleep, and be consoled, rather than the number or severity of withdrawal symptoms which can be subjective. This method has been shown to reduce the need for opioid treatment and shorten hospital stays [4, 9].

The ACT NOW trial used a cluster-randomized, stepped-wedge design, with hospitals randomly assigned times to transition to the Eat, Sleep, Console (ESC) approach. By randomizing at the hospital level but analyzing outcomes at the infant level, the trial could assess individual outcomes like time to discharge while accounting for site-level influences. To address clustering within hospitals, where infants might experience similar outcomes due to shared care practices, the analysis included a site-specific random intercept. This adjustment helped capture differences among hospitals, such as resources, staff expertise, and protocols. Fixed effects for intervention and time were also applied, controlling for the staggered ESC rollout and isolating the intervention’s effect.

These adjustments were essential for detecting variability in ESC’s impact across sites, a phenomenon known as heterogeneity of treatment effect. This variability indicates that ESC may not be equally effective at every hospital, potentially due to differences in implementation, resources, or patient demographics. The trial’s findings suggest that while ESC overall reduced time to discharge, site-specific factors could influence its impact.

For clinicians considering ESC, further insights could come from data on pre-intervention outcomes and how ESC performed in hospitals with similar metrics. While the trial highlighted variability in ESC’s effectiveness across sites, specific pre-intervention baseline data were not included. Additionally, the trial’s challenges highlight the need for hospitals to assess whether they have the resources, training capacity, and family support systems required to implement ESC effectively. By evaluating these factors, hospitals can make more informed decisions on whether ESC is likely to benefit their unique patient population and care environment.

## EBM lesson: stepped wedge design

The stepped wedge design is an approach used in clinical trials suited for evaluating interventions across multiple clusters, such as health services research, in a sequential manner. This design involves a staggered transition where clusters switch from a control condition to an intervention condition at different, randomized time points [10]. Initially, all clusters start in the control state, and progressively, each cluster adopts the intervention according to a pre-determined, randomized sequence. By the end of the study, every cluster has experienced both conditions, allowing for a comprehensive assessment of the intervention’s effectiveness over time [11].

One of the primary advantages of the stepped-wedge, cluster-randomized design is its suitability for settings where randomizing individual participants is not feasible or may disrupt care. In newborn units, as with the ACT NOW trial involving infants with neonatal abstinence syndrome (NAS), randomizing individual infants could interfere with established care practices, making it logistically challenging and ethically complex. By randomizing clusters, such as entire newborn units, rather than individual infants, the trial could introduce the intervention in a way that minimizes disruptions to care while allowing for smoother, systematic implementation across sites.

This design also enhances internal validity by using each cluster as its own control over time, while randomizing the timing of intervention rollout helps control for confounding factors, isolating the effects of the intervention itself. Additionally, the ethical appeal of the stepped-wedge design is noteworthy, as it ensures that all clusters eventually receive the intervention, particularly valuable when the intervention is anticipated to provide benefits.

However, the stepped wedge design introduces several complexities that need to be meticulously managed. One challenge is the complexity of data analysis, as researchers must account for both time-related changes and within- and between-period correlation. This necessitates the use of sophisticated statistical methods to adjust for potential confounding factors and to manage intracluster correlations effectively [12]. Additionally, the design requires careful planning in terms of recruitment and retention, as maintaining the participation of all clusters over an extended period can be difficult [13]. Delays in recruitment or dropout of clusters can adversely affect the study’s power and validity [14]. As Hemming et al. emphasize detailed reporting standards are critical in stepped wedge trials to ensure transparency and reproducibility. Clear documentation of the trial’s design, the rationale for using this approach, and the statistical methods employed are essential for the credibility of the research [15].

A challenge in the ACT NOW trial was the unblinded nature of the stepped-wedge design, a common limitation in cluster-randomized trials. Since providers knew when the intervention was introduced, there was potential for bias, as this knowledge could influence behavior and care practices, introducing expectancy effects or altering standard care during intervention periods.

Traditionally in stepped wedge design, there is a slight preference for the intervention since eventually, all clusters will receive the intervention, however, the equipoise was maintained by systematically evaluating ESC’s safety and effectiveness compared to usual care. This balanced approach was especially important in a vulnerable population like newborns, maintaining both the study’s scientific validity and ethical standards.

In conclusion, the stepped wedge design offers a valuable methodology for evaluating interventions in contexts where traditional RCTs may not be feasible. By balancing ethical considerations with rigorous scientific methods, this design enables researchers to conduct meaningful studies in real-world settings.

## Data Availability

No new data were created or analyzed in this study. Data sharing is not applicable to this article.
